# ﻿A long-read amplicon approach to scaling up the metabarcoding of lichen herbarium specimens

**DOI:** 10.3897/mycokeys.86.77431

**Published:** 2022-02-02

**Authors:** Cécile Gueidan, Lan Li

**Affiliations:** 1 Australian National Herbarium, National Research Collections Australia, NCMI, CSIRO, Canberra, ACT, 2601, Australia Australian National Herbarium, National Research Collections Australia Canberra Australia

**Keywords:** Collection specimens, ITS barcode, lichenised fungi, PacBio amplicon sequencing

## Abstract

Reference sequence databases are critical to the accurate detection and identification of fungi in the environment. As repositories of large numbers of well-curated specimens, herbaria and fungal culture collections have the material resources to generate sequence data for large number of taxa, and could therefore allow filling taxonomic gaps often present in reference sequence databases. Financial resources to do that are however often lacking, so that recent efforts have focused on decreasing sequencing cost by increasing the number of multiplexed samples per sequencing run while maintaining high sequence quality. Following a previous study that aimed at decreasing sequencing cost for lichen specimens by generating fungal ITS barcodes for 96 specimens using PacBio amplicon sequencing, we present a method that further decreases lichen specimen metabarcoding costs. A total of 384 mixed DNA extracts obtained from lichen herbarium specimens, mostly from the four genera *Buellia*, *Catillaria*, *Endocarpon* and *Parmotrema*, were used to generate new fungal ITS sequences using a Sequel I sequencing platform and the PacBio M13 barcoded primers. The average success rate across all taxa was high (86.5%), with particularly high rates for the crustose saxicolous taxa (*Buellia*, *Catillaria* and others; 93.3%) and the terricolous squamulose taxa (*Endocarpon* and others; 96.5%). On the other hand, the success rate for the foliose genus *Parmotrema* was lower (60.4%). With this taxon sampling, greater specimen age did not appear to impact sequencing success. In fact, the 1966–1980 collection date category showed the highest success rate (97.3%). Compared to the previous study, the abundance-based sequence denoising method showed some limitations, but the cost of generating ITS barcodes was further decreased thanks to the higher multiplexing level. In addition to contributing new ITS barcodes for specimens of four interesting lichen genera, this study further highlights the potential and challenges of using new sequencing technologies on collection specimens to generate DNA sequences for reference databases.

## ﻿Introduction

Reference nucleotide sequence databases aim at providing access to curated and high-quality nucleotide sequences representing a broad taxonomic range of living organisms. They are critical to the accurate detection and identification of organisms from environmental samples and, for organisms lacking diagnostic characters, they are a useful tool to confirm morphology-based identifications. In fungi, the internal transcribed spacer region (ITS) has historically been used for species-level molecular identification (Gardes and Bruns 1993; Kõljalg et al. 2005; Abarenkov et al. 2010) and this gene region was later chosen as the fungal universal barcode (Schoch et al. 2012). Well-curated and high-quality fungal ITS sequences are now available from several databases, including RefSeq (Schoch et al. 2014; O’Leary et al. 2015), UNITE (Kõljalg et al. 2013; Nilsson et al. 2018) and ISHAM-ITS (Irinyi et al. 2015). However, the taxonomic coverage for fungi represented in these sequence databases remains incomplete (Orok et al. 2012; Kõljalg et al. 2013; Crous et al. 2014; Nilsson et al. 2018). As well-curated resources of dried or living material of large numbers of fungal species, herbaria and culture collections can contribute to fill some of the taxonomic gaps in these sequence databases (Yahr et al. 2016; Gueidan et al. 2019), and guarantee high taxonomic standards, in particular by prioritising the sequencing of generic and species types (Crous et al. 2014).

Taking advantage of the development of next generation sequencing (NGS) methods, large numbers of fungal ITS sequences have been generated these last ten years. Fungal metabarcoding studies that detect and identify fungi in environmental samples based on inferred operational taxonomic units have mostly generated partial ITS sequences, either ITS1 or ITS2 (Nilsson et al. 2010; Mello et al. 2011; Blaalid et al. 2013). This stems from their preferential use of Illumina sequencing technology which, although allowing affordable high-quality mass molecular barcoding, restricts the maximum read length to 300 bp. Other fungal metabarcoding studies have used long-read technologies, either Roche 454 pyrosequencing (Buée et al. 2009; Lumini et al. 2010; Blaalid et al. 2012; Geml et al. 2014), Pacific Biosciences SMRT sequencing (Chen et al. 2015; Cline and Zak 2015; James et al. 2016; Schlaeppi et al. 2016; Walder et al. 2017; Heeger et al. 2018; Terdersoo and Anslan 2019; Castaño et al. 2020), or Oxford Nanopore MinION sequencing (Hu et al. 2019; Loit et al. 2019) to generate ITS sequences or other molecular barcodes. For lichenised fungi, early long-read metabarcoding studies used Roche 454 pyrosequencing technology (Hodkinson and Lendemer 2013; Lücking et al. 2014; Mark et al. 2016). After the decline of this technology, Pacific Biosciences SMRT sequencing was shown to be a viable option to generate full length high-quality sequences from lichen herbarium specimens (Gueidan et al. 2019).

Although used for whole genome sequencing of lichen metagenomes (Tzovaras et al. 2020), to our knowledge, PacBio SMRT sequencing has only been used for metabarcoding purposes in one study involving lichenised fungi (Gueidan et al. 2019). In this previous study, ITS sequences of 96 lichen specimens were amplified using a two-step PCR approach, with modified ITS primers and PacBio barcoded universal primers. PCR products were then sequenced using the PacBio RS II platform and assembled and denoised using Long Amplicon Analysis (LAA; Bowman et al. 2014). High quality ITS sequences were generated for 88.5% of the samples, with a cost per sample of AU$37. The mixed DNA samples resulting from the DNA extractions of these 96 lichen herbarium specimens also allowed the sequencing of other associated fungi. Here, the same method is used to generate ITS sequences from 384 lichen herbarium specimens, with the main goal of further decreasing the cost per sample. A new set of barcoded universal primers (M13 barcoding system, Larrea et al. 2018) developed by PacBio (Menlo Park, CA, USA) was tested in this study, as well as the then new PacBio Sequel I sequencing platform. Finally, sequence assembly and denoising were performed with a different pipeline, which included SMRT Tools (PacBio, Menlo Park, CA, USA) and DADA2 (Callahan et al. 2016).

The main goals of this study were to 1) assess the current cost and efficiency of a PacBio metabarcoding method applied to lichen herbarium specimens following changes in laboratory and bioinformatic pipelines, and 2) generate high-quality ITS sequences to contribute to reference sequence databases, as well as to molecular taxonomic studies of several lichen groups.

## ﻿Material and methods

### ﻿Taxon sampling and DNA extractions

**Figure 1.**
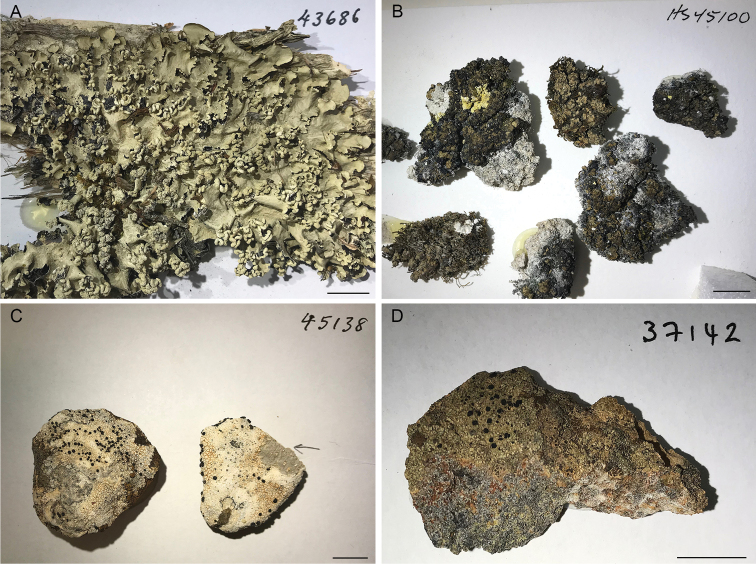
Examples of lichen herbarium specimens used for this study **A***Parmotremaperlatum*, specimen J.A. Elix 43686 (CANB790817) **B***Endocarponpusillum*, specimen H. Streiman 45100 (CBG9011273) **C***Buelliaalbula*, specimen J.A. Elix 45138 (CANB810791) **D***Catillaria* sp., specimen J.A Elix 37142 (CANB872684). Scale bar: 1 cm. Photos C. Gueidan.

For this study, 384 lichen specimens were selected because of their importance to several ongoing taxonomic works on Australian lichens at the Australian National Herbarium (see Suppl. material 1: Table S1). Four main genera were represented (Fig. 1A–D): *Catillaria* (14 specimens from Australia and France), *Buellia* (99 specimens from Australia), *Endocarpon* (167 specimens, including 157 from Australia) and *Parmotrema* (96 specimens from Australia). Additionally, a few other specimens were sampled: *Sporastatia* (4 specimens), and *Halecania* (1 specimen) and 3 unidentified specimens (one crustose saxicolous species and two squamulose terricolous species). The majority of the specimens were identified to the species or genus levels. The specimens were collected between 1966 and 2018, and are kept at CANB, UNSW, NSW, MARSSJ, ABL, BM, HO and in the private herbaria of P. McCarthy, M. Bertrand, B. McCune, and D. Stone. The material of crustose specimens (eg, *Catillaria*, *Buellia*) was detached from the substrate with a clean single-edge razor blade and a weigh paper was used to collect and transfer it to tubes containing a banded ceramic sphere and garnets (2 mL Lysing Matrix A, MP Biomedicals, Seven Hills, NSW, Australia). For squamulose and foliose species (e.g., *Endocarpon*, *Parmotrema*), lobes or squamules were detached from the substrate using clean tweezers and transferred directly to Lysing Matrix A tubes.

The samples were ground with a Precellys Evolution (Bertin Instruments, Montigny-le-Bretonneux, France) in 2–3 cycles of 30 sec at 6,000 rpm. To avoid cross-contaminations, the tubes were briefly centrifuged before the caps were removed. Genomic DNA was extracted using the Invisorb DNA Plant HTS 96 kit (Stratec Molecular, Berlin, Germany) adhering to the manufacturer’s instructions, except for the few following modifications. The lysis buffer and proteinase K were added to each tube of ground material, which were then manually homogenised and incubated at 65 °C for 1 hour. The tubes were centrifuged at 11,000 rpm for 2 min and the supernatants were transferred onto the 96-well prefilter plate using a width-adjustable multichannel pipette. The RNase A (40 µl/well of a 10 mg/ml solution) was added after the prefiltration step and the tubes were incubated at room temperature for 15–20 min before adding the binding buffer. The last centrifugation step was changed to 10 min at 2,000 rpm (instead of 5 min at 4,000 rpm) to avoid breaking the elution plates. The DNA was eluted in 100 µl of elution buffer and 1/10 dilutions of the DNA samples were prepared.

### ﻿Amplification, normalisation and pooling

Indexed PCR products were generated using a 2-step PCR approach as described in the PacBio Barcoded Universal Primers protocol (https://www.pacb.com/wp-content/uploads/2015/09/Procedure-and-Checklist-Preparing-SMRTbell-Libraries-PacB-Barcoded-Universal-Primers.pdf), but with few modifications. The fungal ITS barcode (internal transcribed spacer 1, 5.8S ribosomal RNA subunit and internal transcribed spacer 2) was the target region. With a first PCR, our target region was amplified using the primers ITS1F (Gardes and Bruns 1993) and ITS4 (White et al. 1990), both modified by adding a 5’ block and a tail representing the PacBio M13 primer sequence (Larrea et al. 2018): /5AmMC6/GTA AAA CGA CGG CCA GTC TTG GTC ATT TAG AGG AAG TAA for ITS1F-M13 and /5AmMC6/CAG GAA ACA GCT ATG ACT CCT CCG CTT ATT GAT ATG C for ITS4-M13. In a 25 µl reaction, 5 µl of MyFi buffer (Bioline, London, UK), 1 µl of MyFi polymerase, 2.5 µl of 3 µM of each primer, 13 µl of water and 1 µl of DNA template were added. For each plate, the amplification was done twice, once using the raw DNA extracts and once using a 1/10 dilution of the raw DNA extracts. The 96 PCR reactions were performed in strip tubes with individual caps to avoid cross-contaminations. The PCR program was 5 min at 95 °C, then 20–25 (and occasionally 35) cycles of 30 sec at 95 °C, 30 sec at 53 °C and 1:30 min at 72 °C, followed by a final elongation step for 7 min at 72 °C. Selected PCR products were run onto an agarose gel using the nucleic acid stain GelRed (Biotium, Fremont, CA, USA). If the gel did not show primer dimer bands, no PCR product cleaning was undergone at this stage.

A second amplification was then performed using part of a set of 64 barcoded M13 primers (32 forward and 32 reverse) provided by PacBio (Menlo Park, CA, USA). The barcode sequences were 16 bp long (see Suppl. material 1: Table S2 for their names and sequences). For the second amplification, the PCR products resulting from the raw extracts and the ones resulting from the 1/10 dilutions were first pooled together in order to minimize the number of negative samples. In a 25 µl reaction, 5 µl of MyFi buffer, 1 µl of MyFi polymerase, 2.5 µl of each barcoded primer pairs (3 µM), 13 µl of water and 1 µl of the pooled product of the first round of PCRs were added. The PCR program was 5 min at 95 °C, then 25 cycles of 30 sec at 95 °C, 30 sec at 65 °C and 1:30 min at 72 °C, followed by a final elongation step for 5 min at 72 °C. All PCR products were checked on a gel as previously described and cleaned using AMPureXP beads (Beckman Coulter, Brea, CA, USA). First, the beads were prewashed as recommended for SMRTbell^TM^ library preparation by the Ramaciotti Centre for Genomics (https://www.ramaciotti.unsw.edu.au/sites/default/files/2019-04/RAMAC_Long_Linked_Read_guidelines_2019.pdf). The cleaning was then done by adding 0.8X volume of beads to each PCR product, followed by two washes with 200 µl of 70% ethanol. Dry beads were then resuspended in 25 µl of the EB elution buffer (Qiagen, Hilden, Germany). The concentrations were measured with a Nanodrop 8000 spectrophotometer (Thermo Scientific, Waltham, MA, USA) and 10 ng/µl working solutions were manually prepared. One µl of each of the 384 samples was then pooled into a single tube.

### ﻿Library preparation, sequencing and primary analysis

The pooled sample was sent to the Ramaciotti Centre for Genomics (UNSW Sydney, Australia) for single molecular real-time (SMRT) sequencing. The library preparation was done using the SMRTbell Template Prep Kit v. 1.0 (Pacific Biosciences, Menlo Park, CA, USA). The sample was sequenced in one SMRT cell and with a ten-hour movie, using the Sequel Binding Kit v. 3.0 and the Sequel Sequencing Plate v. 3.0 (Pacific Biosciences). The subread bam file provided by Ramaciotti was generated using SMRT Link v. 6.0 (Pacific Biosciences). This subread bam file was then demultiplexed using the “lima” command in SMRT Tools v. 7.0.1 (Pacific Biosciences), and the circular consensus sequences (CCSs) generated using the “ccs” command (0.9999 minimum predicted accuracy and 3 minimum passes).

### ﻿Secondary analysis and sequence identification

Generated CCSs were denoised using DADA2 v. 1.14 (Callahan et al. 2016), a software that infers sequence variants from high-throughput amplicon sequencing datasets. A custom R script, written by the bioinformatics team at the BRF (ANU, Canberra, Australia) and available upon request, allowed the batch processing of each CCS fastq file through the DADA2 pipeline. First, the primers were removed and the reads reoriented. Read quality profiles and length distribution histograms were then generated for each file. The reads were filtered (maxEE = 1, minQ = 3 and minLen = 300) and dereplicated. The error model was then estimated from the data (BAND-SIZE = 32) and the data further filtered for errors. After a last check for chimeras, fasta files of sequence variants were generated. For sequence identification, a blastn query (BLASTN 2.9.0+) was performed on these files against the NCBI nt database using a max_target_seqs of 3.

The blastn output was parsed into a single text file using a custom script and the results checked manually. Sequencing was considered successful if one of the generated sequence variants matched the same genus as the target taxa. For the unsuccessful samples, the fastq ccs files were converted to fasta using the fastqtofasta command in fastx 0.0.14, and an additional blastn query (BLASTN 2.12.0+) was performed on the ccs files using the same parameter as above. The blastn results were checked manually and sequencing was considered successful if one of the ccs matched the same genus as the target taxa. Demultiplexed fastq files were deposited in the Sequence Read Archive on NCBI (BioProject ID PRJNA796455).

## ﻿Results

### ﻿Amplification and sequencing

The two-step amplification approach generated PCR products with concentrations ranging from 11 to 1,573 ng/µl (Suppl. material 1: Table S1). The average concentration was 90 ng/µl and only 51 of the 384 samples were under 50 ng/µl (Suppl. material 1: Table S1). A total of 1.16 µg of PCR products was submitted to Ramaciotti in a pooled sample. The sample met the quality control requirements and showed DNA fragments ranging from 581 to 1,447 bp, with two clear peaks at 694 and 998 bp, which are within the expected size range for the ITS barcode. The SMART cell generated 217,195 polymerase reads, with a mean length of 52,801 bp. This corresponded to 12,955,790 subreads with a mean length of 841 bp, and an average of 60 passes per ccs.

### ﻿Sequence analysis and sequence identity

Using SMRT Tools, CCSs were recovered for 372 of the 384 samples, with only 12 samples for which no reads were generated (Suppl. material 1: Table S1). For each positive sample, between 22 and 1,368 CCSs were generated. After sequence denoising using the DADA2 amplicon pipeline, 1 to 21 sequence variants were found per sample. A blast analysis was conducted on all sequence variants and the results were compared to the morphology-based genus and/or species identification. Following this denoising step, a sequence of the target taxa was generated for 262 of the 384 initial samples. For 70 samples, no sequence variant matching the target taxon was generated by DADA2, but one or more sequences of the target taxon could be recovered from the CCS files. Therefore, in total, 332 of the 384 initial samples were considered as successful (86.5%). For 40 samples, sequence of the target taxon could not be found neither amongst the sequence variants generated by DADA2, nor among the CCSs. The majority of the samples that failed to generate sequences from the target species were from specimens of the genus *Parmotrema* (33 samples out of 40, or 82.5%).

**Figure 2.**
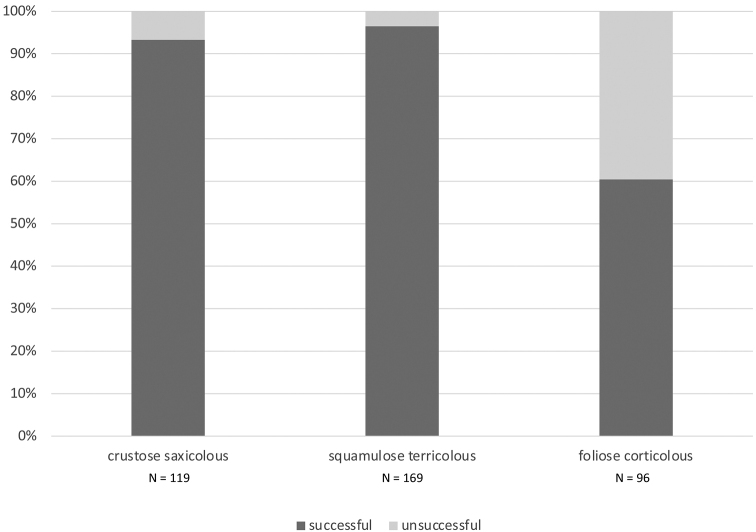
Sequencing success for different morphological groups of taxa included in this study. Specimens were grouped into three main morphological categories: **1***Buellia*, *Catillaria* and other crustose saxicolous taxa **2***Endocarpon* and other squamulose terricolous taxa **3** the foliose corticolous genus *Parmotrema*. In the graph, stalked columns show successful samples (sequence generated for the target species) in dark grey and unsuccessful samples (no sequence generated or generated sequences not from the target species) in light grey. The total number of samples (N) is indicated below each corresponding column.

**Figure 3.**
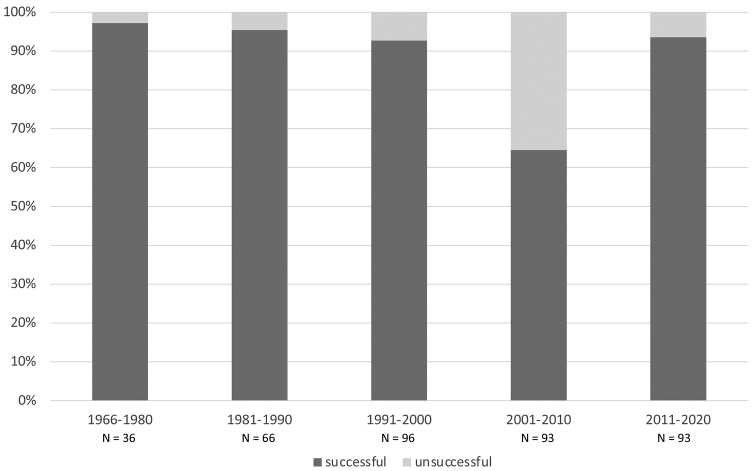
Sequencing success for different ages of specimens included in this study. Specimens were grouped in five categories: 1966–1980, 1981–1990, 1991–2000, 2001–2010, 2011–2020. In the graph, stalked columns show successful samples (sequence generated for the target species) in dark grey and unsuccessful samples (no sequence generated or generated sequences not from the target species) in light grey. The total number of samples (N) is indicated below each corresponding column.

When divided into three main morphological groups of taxa (Fig. 2), *Buellia*, *Catillaria* and other saxicolous crustose taxa had a sequencing success rate of 93.3% (111 positive samples out of 119). *Endocarpon* and other terricolous squamulose taxa had a sequencing success rate of 96.5% (163 positive samples out of 169 sample). Finally, the foliose genus *Parmotrema* had a sequencing success rate of only 60.4% (58 positive samples out of 96). When divided in five categories of time of collection (1966–1980, 1981–1990, 1991–2000, 2001–2010 and 2011–2020), the proportion of unsuccessful samples does not increase with the age of specimens: all but one category had more than 90% success rate (Fig. 3). In fact, the highest success rate was for the oldest class of specimens, 1966–1980, with 97.2%. The lower success rate for the 2001–2010 class (64.5%) was due to the lower success rate of specimens of *Parmotrema*, most of which were collected between 2005 and 2010.

## ﻿Discussion

Building upon a previous work (Gueidan et al. 2019), the goal of this study was to generate high-quality ITS sequences for 384 lichen herbarium specimens and assess the current efficiency and cost of a modified PacBio metabarcoding method that had previously been applied to lichen herbarium specimens.

### ﻿Generation of ITS barcodes for 384 lichen herbarium specimens

ITS sequences were successfully generated for 332 of the 384 herbarium specimens included in this study. Most of the specimens included belonged to the four genera *Buellia*, *Catillaria*, *Endocarpon* and *Parmotrema*. The success rate for the sequencing of the target ITS barcode was high (an average of 86.5% across all taxa) and similar to the one reported in Gueidan et al. (2019), which was of 88.5%. The sequencing success rate for the genus *Parmotrema* was, however, particularly low (60.4%), especially in comparison to *Buellia*, *Catillaria* and other crustose taxa (93.3%) and *Endocarpon* and other squamulose taxa (96.5%). Although a PCR product was obtained for all the *Parmotrema* samples, generated ITS sequences did often not belong to the target species, but to other fungi. This is likely due to inefficient amplification of the ITS barcode from the target *Parmotrema* species using the ITS1F-ITS4 primer pair. Although this primer pair is commonly used to amplify ITS of lichenised fungi (e.g., James et al. 2006; Kelly et al. 2011; Mark et al. 2016), it seems to have been used in combination with one other primer pair, ITS1-LM (Myllys et al. 1999) and ITS2-KL (Lohtander et al. 1998), in previous studies on the genus *Parmotrema* (Divakar et al. 2005; Del Prado et al. 2011). This indicates a possible amplification issue with the ITS1F-ITS4 primer pair for these species. Further sequencing will be carried out in the future for this genus using the alternative primer pair ITS1-LM/IT2-KL, to attempt recovering ITS barcodes for additional *Parmotrema* species.

The genomic DNA of some groups of lichens, most often from crustose corticolous tropical families (e.g., Staiger et al. 2006 for the Graphidaceae; Weerakoon et al. 2012 and Gueidan et al. 2016 for the Pyrenulaceae), is notoriously difficult to obtain from dried specimens. However, for most lichens, ITS sequences can usually easily be obtained from extracts of a large range of lichen herbarium specimens, including some relatively old ones. The oldest ones were a 75-year-old specimen of *Aspiciliaaschabadensis* (Sohrabi et al. 2010), a 100-year-old specimen of *Staurolemmaomphalarioides* (Bendiksby et al. 2014), and a 151-year-old specimen of *Caloplacaconversa* (Redchenko et al. 2012). A sequence of the small subunit of the mitochondrial ribosomal RNA gene (mtSSU) was also obtained from a 127-year-old specimen of *Peltigeracollina* (Kistenich et al. 2019). In the latter study, which used Ion Torrent sequencing to generate mtSSU sequences from historical lichen specimens collected from 1885 onwards, specimen age had a significant influence on sequencing success, with older specimens less likely to yield good quality sequences (Kistenich et al. 2019). In our study, ages of lichen herbarium specimens ranged from 2 to 54 years. Apart from the specimen age category which included most *Parmotrema* samples (2001–2010), for which a primer issue caused low sequencing success, all age categories had high sequencing success rates, ranging from 92.7% to 97.2% (Fig. 3). Therefore, at least for some of the represented groups (*Endocarpon* and saxicolous *Buellia* and *Catillaria*), generating ITS sequences for specimens up to 50 years old was not an issue. These results highlight the importance and relevance of older herbarium specimens, including types, in molecular taxonomy.

### ﻿Efficiency and cost of the applied method

PacBio long read sequencing is a powerful approach, which when applied to amplicons, can utilise circular sequencing to generate high quality consensus of shorter nucleotide fragments. In order to correct sequencing errors, subreads extracted from one polymerase read – therefore generated from a single amplicon molecule, are aligned and assembled into one circular consensus sequence (CCS). Following CCS generation, additional software and pipelines are available to further correct sequencing errors, a step often called denoising. Although several software are available for denoising Illumina amplicon data (e.g., unoise, Edgar 2016; deblur, Amir et al. 2017), very few are available for PacBio amplicon data. In a previous study (Gueidan et al. 2019), a denoising approach developed for allele phasing of PacBio amplicon data (LAA) was used to generate high quality ITS sequences from lichen herbarium specimens. Parallel Sanger sequencing for a subset of samples confirmed that this method worked well for generating high-quality sequences for target and associated fungal species (Gueidan et al. 2019). However, a more relevant approach for metabarcoding is available (DADA2, Callahan et al. 2016) and was tested here. DADA2 is a software package that allows to generate amplicon sequence variants from high-throughput amplicon sequencing data. Conveniently, it can use PacBio data and a protocol is available for the size-variable ITS marker.

Despite DADA2 generating target sequence variants for a large number of our samples, a significant number of samples (70) did not yield sequences from the target taxon despite having one to several CCSs that matched the target taxon. For error correction, DADA2 is trained on a pool of sequences and uses sequence abundance to discriminate between sequencing error and true sequence variation. In our case the sequence pools corresponded to each of the 384 samples and were rather small due to the high level of multiplexing (average of 229 CCSs per sample/pool). In addition, among the CCSs available for each pool, in particular for the samples for which DADA2 did not recover the target taxon, the target CCSs were in low abundance within a large pool of lichen-associated fungal sequences or contaminant sequences. Because DADA2 error correction is based on sequence abundance, sequence variants are only inferred for high-abundance sequences. It is therefore not fully applicable to the metabarcoding of lichen herbarium specimens, or at least not when sequences of associated fungi are abundant. In this case, denoising methods that are not based on sequence abundance may perform better.

In terms of sequencing efficiency, with an average sequencing success rate of 86.5%, the new M13 amplicon sequencing protocol from PacBio is comparable to the protocol used in Gueidan et al. (2019). More recently, a UMI-based protocol for both Nanopore and PacBio long-read sequencing, which further decreases chimera and error rates, has been developed (Karst et al. 2021) and would be worth testing as well in the future. In terms of time, both plate DNA extractions and batch sequence editing allowed us to decrease the time necessary to obtain the final ITS barcode sequences. The method also allows eliminating the time-consuming cloning step for samples with co-amplified fungal products. In terms of cost, the present method allowed us to decrease the cost per sample from AU$37 (Gueidan et al. 2019) to AU$27 (this study). The cost reduction is less than what was anticipated in Gueidan et al. (2019), mostly due to the transition from the RSII sequencing platform to the Sequel sequencing platform, which is more expensive. It is also due to the initial cost of a plate of M13 PacBio universal barcoded primers, which was more expensive than the Barcoded Universal F/R Primers Plate-96 from PacBio used in Gueidan et al. (2019). The M13 PacBio universal barcoded primer plate, however, includes larger volumes and can be used for more reactions. Similarly, although more expensive, the Sequel platform generates a much larger data output than the RSII platform (5–11 Gb versus 500 Mb, respectively). Sequel II, the next generation of SMRT sequencing platform, generates an even larger output (about 80 Gb), while the RSII platform is currently being discontinued in most sequencing services. These changes to the SMRT sequencing platforms and protocols imply that, at present, for lichen specimen metabarcoding, only pooled samples of large numbers of specimens (>500) or larger number of markers will make this technology cost-efficient.

## ﻿Conclusion

With an average sequencing success of 86.5%, this long-read amplicon sequencing method is confirmed as a potential alternative to Sanger sequencing for the generation of full-length and high-quality DNA barcodes from mixed DNA samples extracted from lichen specimens. It performed particularly well for crustose saxicolous (93.3% success) and squamulose terricolous (96.5% success) taxa. In terms of cost (AU$27/sample), although still more expensive than Sanger sequencing, it allows recovering high-quality sequences even when other lichen-associated fungi amplify as well, eliminating the need for using gel separation or cloning. At high multiplexing level (more than 500 samples/run), this high-throughput method is therefore an attractive option for the generation of DNA barcodes from large number of herbarium specimens.

## Supplementary materials

Supplementary material 1 Table S1. List of specimens used for this study, including their voucher information, plate location, indexing, amplicon concentration and sequencing results, both as an output from SMRT tools (CCSs) and as an output from DADA2 (sequence variants). Table S2. List of the 64 barcode sequences used to index the samples. Used barcode pairs are listed in Table S1 Link: https://doi.org/10.3897/mycokeys.86.77431.suppl1 Data type Taxon sampling Explanation note List of specimens used for this study, including their voucher information, plate location, indexing, amplicon concentration and sequencing results, both as an output from SMRT tools (CCSs) and as an output from DADA2 (sequence variants). A summary of the blast results for the sequence variants is also listed for each sample. In the "recovered target" column, samples for which the target sequence was recovered from the CCS file but not the sequence variant file are indicated by a star. https://binary.pensoft.net/file/641123This dataset is made available under the Open Database License (http://opendatacommons.org/licenses/odbl/1.0/). The Open Database License (ODbL) is a license agreement intended to allow users to freely share, modify, and use this Dataset while maintaining this same freedom for others, provided that the original source and author(s) are credited.Cécile Gueidan, Lan Li
